# Impact of shredding degree on papermaking potential of recycled waste

**DOI:** 10.1038/s41598-021-96325-4

**Published:** 2021-09-01

**Authors:** Aneta Lipkiewicz, Edyta Małachowska, Marcin Dubowik, Piotr Przybysz

**Affiliations:** 1 Natural Fibers Advanced Technologies, 42A Blekitna Str., 93-322 Lodz, Poland; 2 Institute of Wood Sciences and Furniture, Warsaw University of Life Sciences - SGGW, 159 Nowoursynowska Str., 02-787 Warsaw, Poland

**Keywords:** Ecology, Environmental sciences, Engineering, Materials science

## Abstract

The properties of paper products depend on the structure of the cellulose fibres therein. Although fibre properties in virgin pulps can be modified by a refining process, this is more difficult in pulp from recovered fibre, particularly waste from office shredders that tend to shorten fibres during shredding. The shorter fibres in shredded paper make it difficult to easily reconstitute them into high-quality paper products. Moreover, because of high energy usage during the recycling process and transportation inefficiencies, there is a need to determine how to responsibly shred paper to alleviate this environmental burden. With this in mind, the influence of initial fibre length on the tensile properties of paper was investigated. Changes in initial fibre length significantly influenced many pulp and paper properties. It was found that cutting the paper into pieces with an area less than 25 mm^2^ caused significant changes in the important morphological parameters of the fibres and a sharp decrease in the tensile properties of the reconstituted paper.

## Introduction

Wastepaper, for both ecological and economic reasons, is a good raw material for the production of paper or cardboard. Paper recycling reduces the use of wood, and thus, helps preserve forest resources, saves energy, reduces littering, the amount of waste going to landfills, air pollution, and wastewater generation, and instils ecological attitudes in society.

However, the properties of fibres in wastepaper can deteriorate not only during processing, but also at the collection stage due to shredding, which can cause excessive shortening of the fibres. In addition to fibre bonding^1^, fibre length and strength are basic factors influencing the tensile and structural properties of paper products^2–4^. Fibre and pulp properties also affect the cost of producing paper products. Hence, the ability to control fibre properties during the recycling stage is a determining factor in effective quality control and the cost of paper production from waste paper.

In industrial practice, the fibres are shortened as a direct result of the fibre refining process or by high-shear processing of the fibrous suspension in the refining zone^5,6^. Therefore, the process of pulp refining has a direct influence on fibre properties, and consequently the properties of the final product^7–9^. Through refining, the properties of the refined pulp can be modified to obtain paper with the desired properties. The refining process, aside from affecting paper properties, also has a decisive impact on the unit energy consumption in this process^10–13^. Owing to the increasing global growth of the market for paper products^14,15^, it is extremely important to minimise the unit energy consumption in this process and optimise the development of useful properties of the paper during processing.

Changes taking place in the structure of the refined fibres during the refining process determine how the pulp behaves during web formation and the basic properties of the paper that is produced^16,17^. Fibre shortening has a direct negative impact on the dynamic properties of paper, including its tear resistance^18,19^. Often, refining is consciously carried out to improve the conditions of web forming and improve its transparency.

When paper is thrown into shredders, the objective is to destroy documents; however, the process also unconsciously shortens the fibres. Paper shredders should facilitate the preparation of pulped materials for further production, rather than render the paper useless. Cut fibres in the shredded paper make it difficult to carry out easy reconstitution into high-quality paper products. Moreover, most recycling centres do not handle small strips or bits of paper. Large-scale recycling facilities use large screens to dry pulped paper on, and finely shredded paper is not well retained and can fall through the screens. Reports claim that the utilisation or recycling of shredded paper is much more problematic than that of mixed unshredded paper. The Environmental Paper Network, a worldwide association of 140 civil society groups and NGOs concerned with the sustainability of pulp and paper practices suggests shredding paper only when necessary^20^, especially since destruction also increases the bulk density of the paper, reducing its transportation efficiency. The office waste problem is particularly significant because of the high amount generated. For example, Japan has a paper collection rate of around 81.6%^21^, which is higher than that of other countries. However, this high collection rate is mainly constituted by a high recycling rate for paper grades such as cardboard and newspapers. The collection rate for office paper and shredded paper remains low (less than 60%)^21^ because office paper, on which confidential information is often printed, is generally disposed of. In U.S. offices, 50% of business waste is composed of paper. Offices use approximately 12.1 trillion sheets of paper per year, and paper accounts for 25% of landfill waste and 33% of municipal waste. It was found that each tonne of recycled paper allows for 64% energy savings, 58% water savings, and 60 pounds less air pollution^22^. Security issues aside, one should consider how to efficiently destroy documents while still allowing fibres to be efficiently used in further processing, which will enable the above savings; this paper attempts to address this issue.

Paper destruction categories are assigned based on the standard German Deutsches Institut für Normung (DIN) classification for paper shredding machines. DIN 66,399 classifications, in which ‘P’ refers to ‘Paper-Based’ material, are based on the size and type of particle (Table 1).

**Table 1:** Requirements for machines and processes shredding documents (DIN 66399).

Specific security levels	Paper media requirements
P-1	12 mm strips or maximum particle surface area of 2.000 mm^2^
P-2	6 mm strips or maximum particle surface area of 800 mm^2^
P-3	2 mm strips or maximum particle surface area of 320 mm^2^
P-4	Maximum cross cut particle surface area 160 mm^2^ with a maximum strip width of 6 mm = 6 × 25 mm
P-5	Maximum cross cut particle surface area 30 mm^2^ with a maximum strip width of 2 mm = 2 × 15 mm
P-6	Maximum cross cut particle surface area 10 mm^2^ with a maximum strip width of 1 mm = 1 × 10 mm
P-7	Maximum cross cut particle surface area 5 mm^2^ with a maximum strip width of 1 mm = 1 × 5 mm

The aim of this study is to examine the effect of the surface area of shredded particles on paper properties. The study therefore estimates the extent to which waste paper can be shredded while still producing a high-quality product. Pulp samples with different initial particle sizes treated under constant papermaking conditions were used for this purpose.

## Materials and methods

### Sample preparation

Industrial air-dried and bleached kraft pine pulp in the form of sheets (Arctic Paper Kostrzyn S. A.) was used in this study. In order to keep all other parameters constant, samples of the pulp were cut manually into squares of different areas (1–400 mm^2^) to reduce and diversify the fibre length.

Hand-cut samples were compared with strips from shredding machines (destroyed in accordance with DIN 66399). The following shredders were used for the tests: Kobra 240.1 S2 ES (for strips with P-3 specific security levels), HSM Shredstar S5 (for strips with P-4 specific security levels), HSM Securio C18 (for strips with P-5 specific security levels), and HSM Securio B26 (for strips with P-6 and P-7 specific security levels). All cutting processes in the shredders were performed without the addition of oil.

### Production of paper sheets and analysis of pulp and paper properties

Sheets of paper from the cut samples were produced under laboratory conditions from rewetted pulp samples (22.5 g dry weight samples were soaked in water for 24 h) that were subjected to disintegration using a laboratory JAC SHPD28D propeller pulp disintegrator (Danex, Katowice, Poland) for 23.000 revolutions, according to ISO 5263-1 (2004). The disintegrated pulps were concentrated to a dry weight content of 10% and refined in a JAC PFID12X PFI mill (Danex, Katowice, Poland) under standard conditions [ISO 5264-2 (2011)]. All the samples were refined for a constant time of 120 s. After the model pulp recycling processes, including refining, the following properties of the pulps were evaluated:

- Schopper-Riegler freeness parameter (SR) was measured using a Schopper–Riegler apparatus (Danex, Katowice, Poland) in accordance with PN-EN ISO 5267-1 (2002);
- The water retention value (WRV) was determined according to ISO 23714 (2014);
- The dimensions of the fibre parameters were measured according to ISO 16065-2 (2016) using a Morfi Compact Black Edition apparatus (Techpap, Grenoble, France).

In the next step, sheets of paper were formed in a Rapid-Koethen apparatus in accordance with PN-EN ISO 5269-2 (2007). Each paper sheet had a basis weight of 80 g/m^2^ (according to ISO 536:2012). Only sheets with basis weights between 79 and 81 g/m^2^ were used for further investigation. The sheets were conditioned for 24 h at a relative humidity of 50 ± 2% and a temperature of 23 ± 1°C [ISO 187 (1990)] before determining their properties. The properties of the paper sheets were examined as follows. A ZwickRoell Z005 TN ProLine tensile testing machine (Zwick-Roell, Ulm, Germany) was used to measure the mechanical properties of the paper in accordance with PN-EN ISO 1924-2 (2010). The roughness and air permeability were measured using a Bendtsen apparatus (Messmer Buchel, Veenendaal, The Netherlands).

The average degree of polymerisation (DP) of cellulose in each pulp was determined using the viscometric method described in ISO 5351 (2010).

## Results and discussion

In industrial conditions, the freeness index of pulp is a commonly used parameter to assess the degree of refining of pulp based on how easily it dewaters. The dewatering of pulp on a paper machine screen is a very useful indicator in industrial practice; therefore, in this study, the effect of the initial fibre length on changes in this indicator was examined. Table 2 shows that the freeness of the refined pulp decreases as the initial fibre length increases, which is attributed to the decreasing amount of fine material in the pulp, in line with the current state of knowledge^23–28^. It is assumed that a freeness index of about 30°SR is optimal for most papermaking properties^29,30^. However, obtained results do not indicate that a freeness of ~ 30°SR necessarily achieves a maximum tensile strength (Table 2). This confirms that there is no straightforward relationship between freeness and paper properties. Therefore, the freeness of the pulp is not useful to compare the papermaking potential of pulp with different initial parameters.

**Table 2:** Characteristics of refined cellulose pulps.

Sample dimensions (mm)	Sample area (mm^2^)	Freeness	WRV
		(°SR)	(%)
1 × 1	1	51	214.5 (3.4)
2 × 2	4	42	212.5 (2.2)
1 × 5 (P-7)	5	39	211.1 (1.2)
3 × 3	9	31	208.5 (1.6)
1 × 10 (P-6)	10	31	208.1 (1.3)
4 × 4	16	30	207.2 (1.4)
5 × 5	25	29	206.8 (1.2)
2 × 15 (P-5)	30	28	206.3 (1.5)
6 × 6	36	28	206.1 (1.9)
8 × 8	64	26	205.2 (2.5)
10 × 10	100	25	205.6 (1.8)
6 × 25 (P-4)	150	25	204.8 (2.2)
P-3	320	25	203.6 (2.8)
20 × 20	400	25	202.8 (1.6)
Reference	–	25	201.2 (3.1)

Standard deviation are given in brackets.

The impact of initial fibre length on internal fibrillation, one of the basic effects of refining^31–35^, was also studied. Progress in achieving internal fibrillation of refined fibres is commonly assessed based on an increase in their swelling^36,37^, usually measured by the WRV^38–40^. The analysis in Table 2 indicates that the WRV increases as the initial fibre length decreases, reaching a maximum value of 214.5%. This increase in fibre swelling is accompanied by an increase in the density of the paper, which in turn increases the resistance of the paper to air permeability (Table 2), consistent with previous studies^8,41–43^.

The initial fibre length did not affect the DP of the produced pulps. The DP was 931 ± 0.89 regardless of the initial dimensions of the samples tested. This confirms previous findings in the literature that mechanical treatment has little effect on the DP of cellulose^44,45^.

**Figure 1:**
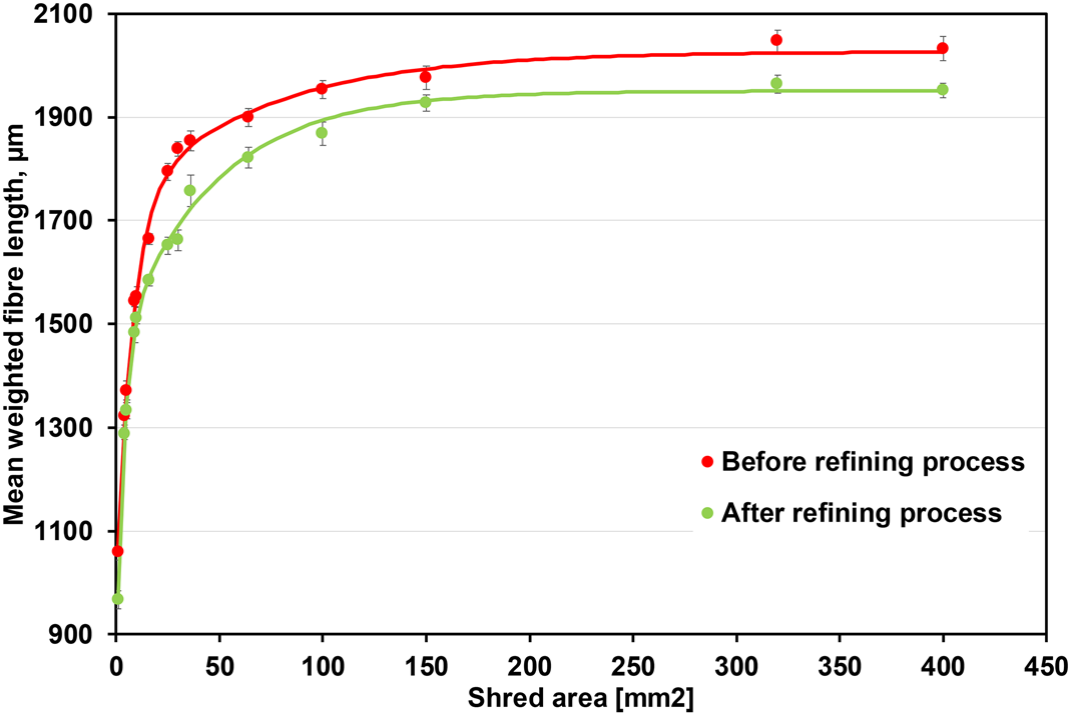
Average fibre length dependency on the cut surface area of samples (before and after model pulp recycling process including refining).

Table 3 and Fig. 1 show the morphological characteristics of the fibres of the examined pulps. The fibre length for the refined pulps is characterised by lower values compared to the unrefined pulps, which confirms that one of the basic effects of refining is fibre shortening^46–48^. It should be noted that the use of mean weighted or mean geometric fibre length eliminates the influence of the fines fraction on the analysis result^49,50^.

**Table 3:** Fibre and pulp properties after model pulp recycling process including refining.

Sample area	Mean fibre width	Mean fibre coarseness	Macro fibrillation index	Broken fibre content	Fine content
mm^2^	µm	mg/m	%	%	% in area
1	32.9 (0.1)	0.207 (0.017)	1.135 (0.047)	52.3 (1.1)	11.70 (0.54)
4	32.4 (0.3)	0.199 (0.009)	1.085 (0.007)	50.0 (0.5)	9.42 (0.13)
5	32.5 (0.2)	0.197 (0.008)	1.059 (0.009)	49.3 (0.6)	9.20 (0.19)
9	31.9 (0.1)	0.190 (0.011)	0.890 (0.023)	45.8 (0.4)	6.85 (0.24)
10	31.8 (0.2)	0.195 (0.007)	0.903 (0.020)	46.1 (0.3)	6.78 (0.18)
16	31.5 (0.1)	0.196 (0.006)	0.861 (0.011)	44.9 (0.8)	5.98 (0.18)
25	31.7 (0.4)	0.195 (0.001)	0.832 (0.007)	43.8 (1.5)	5.51 (0.14)
30	31.5 (0.3)	0.193 (0.002)	0.819 (0.006)	43.6 (1.1)	5.26 (0.16)
36	31.2 (0.2)	0.191 (0.008)	0.814 (0.005)	43.0 (0.6)	5.08 (0.28)
64	31.5 (0.1)	0.187 (0.009)	0.799 (0.012)	41.5 (0.2)	4.26 (0.14)
100	31.1 (0.1)	0.182 (0.012)	0.764 (0.022)	39.3 (0.6)	3.25 (0.21)
150	31.0 (0.3)	0.184 (0.011)	0.769 (0.015)	39.8 (0.4)	3.04 (0.18)
320	30.8 (0.1)	0.183 (0.010)	0.752 (0.012)	38.4 (0.4)	2.95 (0.10)
400	30.9 (0.4)	0.184 (0.001)	0.758 (0.058)	39.2 (0.5)	3.00 (0.60)
Reference	30.7 (0.3)	0.180 (0.010)	0.777 (0.024)	35.4 (0.6)	2.81 (0.60)

Standard deviation are given in brackets.

The results presented in the Table 3 show that the examined parameter values of fibres and pulps (mean fibre width, mean fibre coarseness, macro fibrillation index, broken fibre content, and fine content) tend to decrease with decreasing length of the initial fibres, and consequently, the mean weighted fibre length increases. However, an initial sample length greater than 5 mm does not significantly affect the examined fibre properties and fine content. It is therefore likely that a strip width of 5 mm is the limit above which significant changes in the pulp do not occur. The results are similar before and after the refining process (Fig. 1).

**Figure 2:**
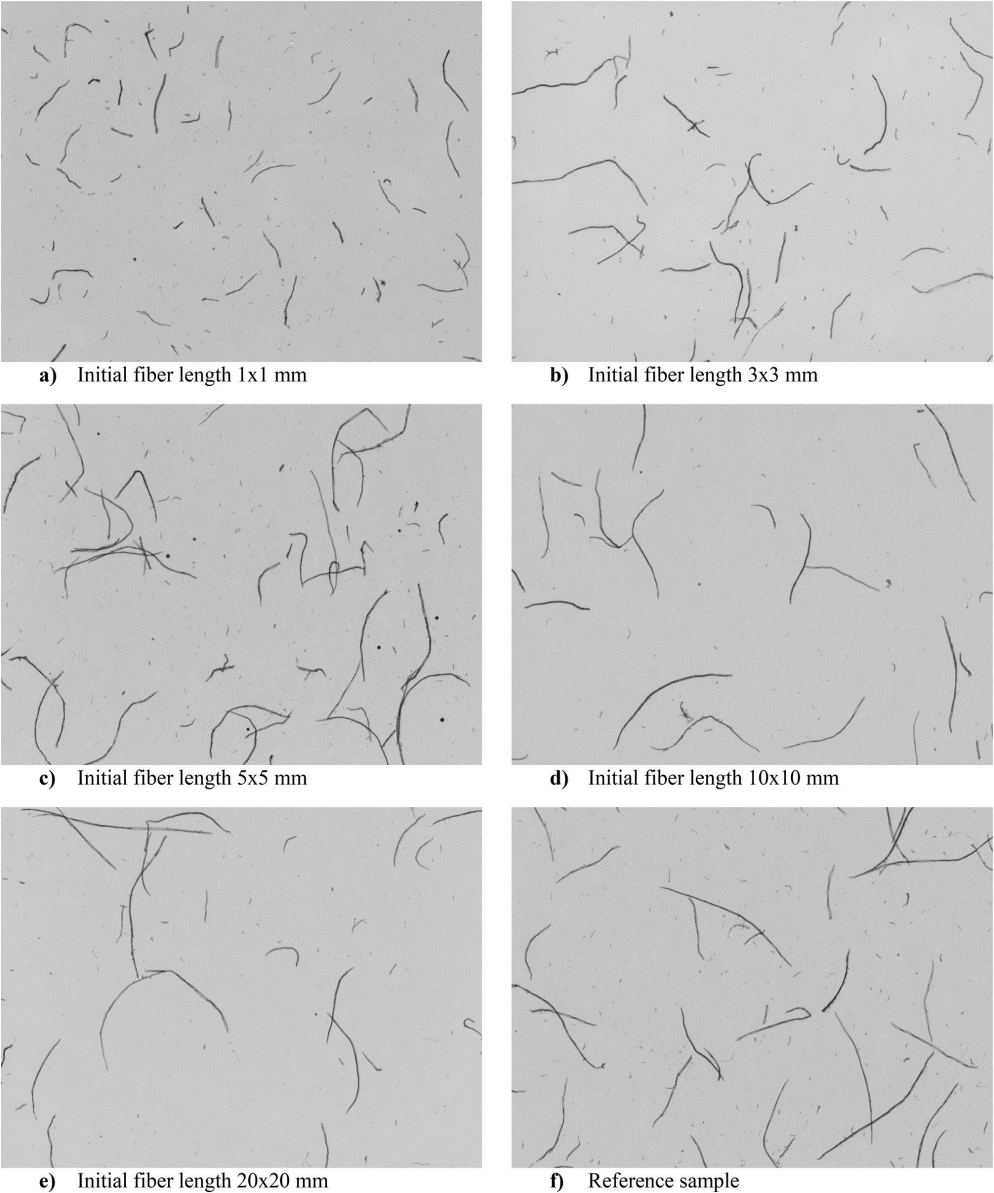
Microscopic images of tested fibrous materials.

Microscopic images of the refined pulps, recorded using a Morfi Compact Black Edition camera, are shown in Fig. 2. The decrease in fibre length after the model pulp recycling process is proportional to the decrease in the dimensions of the pre-cut pulp samples. The most significant fibre shortening is noticed for the 1 × 1 mm sample. There is no significant difference in fibre dimensions for the 10 × 10 mm samples and the reference sample (Fig. 2). Therefore, it is possible to shred paper in a shredder to a specific level of fragmentation without fear of excessive shortening of the fibres, which would make the production of high-quality paper more difficult.

According to previous findings, fibre length has a significant impact on the paper stretch index^51^, and excessive fibre shortening and a high fines content cause the paper product to become rigid and reduce its deformability^49,52,53^. Our results, however, indicate that the stretch of the examined papers is similar, irrespective of the fibre length and fines fraction content (Table 4), in contrast to previously reported results. However, the results listed in Table 4 show that fibre length exerts a significant impact on the dynamic tensile properties of paper^54–56^. Importantly, in the case of fibre properties (Table 3 and Fig. 1), changes in the area of the shredded samples above 25 mm^2^ did not significantly affect the tensile paper properties (Table 4). Based on this, it can be concluded that samples can be cut at 5 mm or larger without significant shortening of the fibres and no significant changes in pulp and paper properties. Therefore, the paper shredding provides useful wastepaper when performed in devices up to class P-6.

**Table 4:** Mechanical properties of paper sheets produced from refined pulps.

Sample area	Force at break index	Stretch	Energy absorption index	Tensile stiffness index	Roughness	Air permeability
mm^2^	Nm/g	%	J/g	Nm/g	ml/min	ml/min
1	47.7 (0.8)	4.38 (0.01)	1.53 (0.07)	5116 (134)	230 (5)	156 (2)
4	57.9 (1.0)	4.43 (0.05)	1.80 (0.05)	5813 (146)	243 (3)	256 (2)
5	58.7 (0.9)	4.42 (0.03)	1.77 (0.04)	5866 (132)	251 (5)	252 (3)
9	69.0 (0.6)	4.30 (0.09)	2.05 (0.04)	5969 (118)	263 (8)	527 (4)
10	68.8 (0.5)	4.39 (0.08)	2.11 (0.03)	5963 (122)	269 (7)	533 (6)
16	70.6 (0.7)	4.21 (0.05)	2.15 (0.04)	6124 (147)	281 (6)	629 (3)
25	72.6 (1.1)	4.53 (0.02)	2.23 (0.05)	6334 (154)	290 (10)	857 (5)
30	73.2 (1.0)	4.44 (0.03)	2.20 (0.03)	6371 (139)	298 (8)	876 (6)
36	73.5 (0.3)	4.38 (0.06)	2.18 (0.02)	6384 (111)	301 (3)	954 (4)
64	74.0 (0.5)	4.23 (0.03)	2.24 (0.03)	6412 (125)	304 (7)	1201 (10)
100	74.5 (0.7)	4.45 (0.04)	2.20 (0.05)	6301 (108)	321 (8)	1315 (9)
150	74.6 (0.3)	4.36 (0.04)	2.16 (0.01)	6358 (111)	343 (5)	1354 (7)
320	74.6 (0.3)	4.42 (0.03)	2.19 (0.02)	6294 (130)	339 (6)	1418 (11)
400	74.5 (0.6)	4.32 (0.03)	2.15 (0.07)	6215 (149)	351 (10)	1415 (13)
Reference	74.6 (0.9)	4.61 (0.03)	2.20 (0.04)	6344 (151)	344 (8)	1421 (22)

Standard deviation are given in brackets.

Research has shown that the fines fraction produced from fibres is responsible for slowing pulp-dewatering in the forming section of a paper machine^57,58^. The results obtained are fully consistent with those of earlier studies, in that the pulps with the highest fines content also have the highest SR freeness values (Table 2).

The air permeability of paper decreases as the SR freeness level increases^59–62^, in agreement with previous results. The pulps characterised by lower average fibre length, and therefore, have improved barrier properties to gases even though their tensile properties are reduced (Table 4).

The roughness of paper increased with increasing initial fibre length, in agreement with previous results^34,61,63,64^. Therefore, the best smoothness results (230 mL/min) were obtained for paper produced from the pulp with the lowest fibre length. From the data provided, it can be concluded that this paper would likely have the best printability.

**Figure 3:**
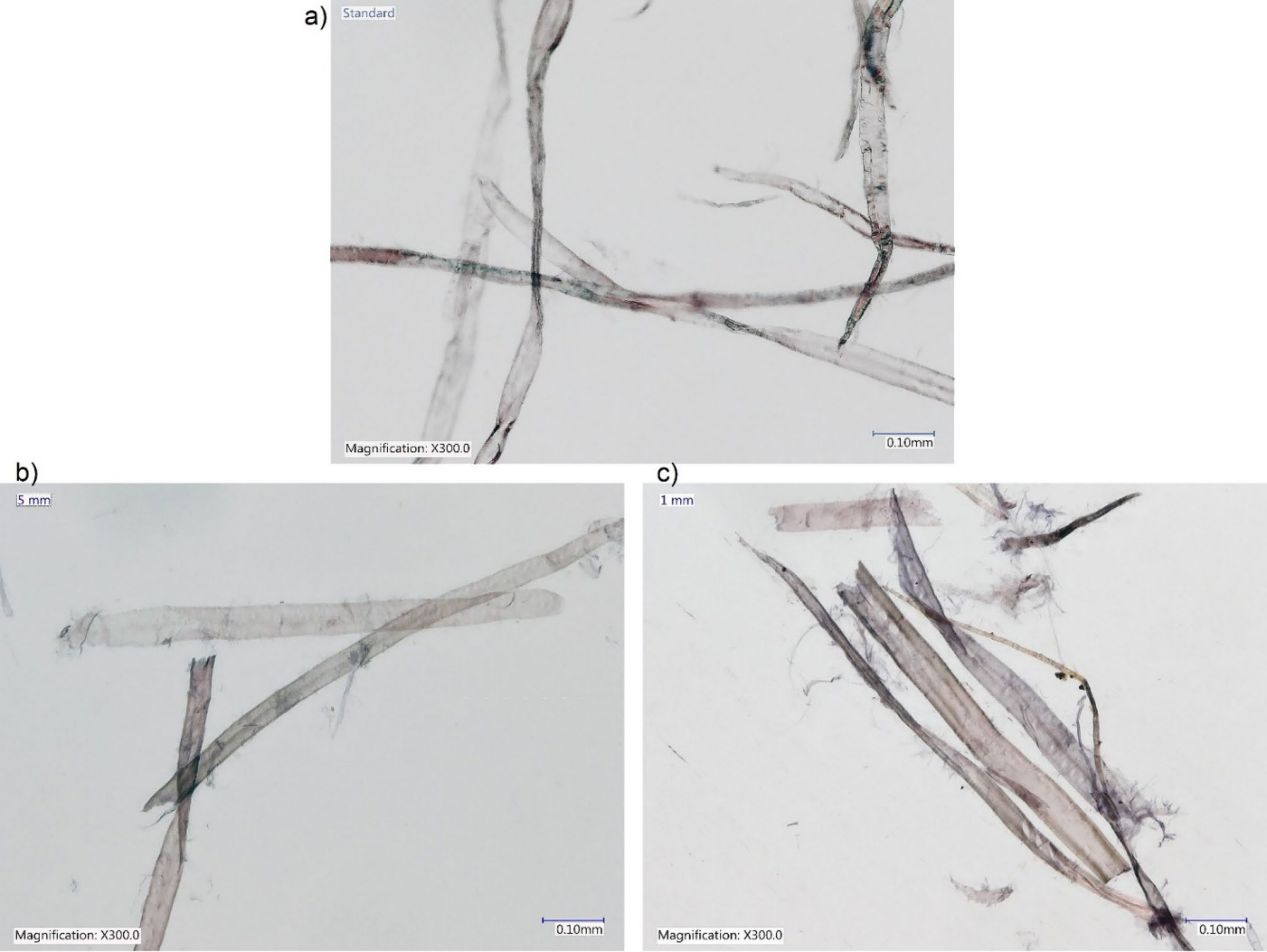
Microscopic images of paper sheets derived from (**a**) reference and (**b**, **c**) selected refined pulps produced from the tested fibrous materials.

Microscopic images of the paper sheets, recorded using a Keyence VHX-6000 microscope equipped with a VH-Z100UR lens, are presented in Fig. 3. The fibres in the reference pulp (Fig. 3a) appear undamaged. The paper obtained from samples cut into 5 × 5 mm pieces (Fig. 3b) shows both undamaged fibres and cut fibres. This observation clearly indicates that initial shortening of fibres damages the fibre structure to some extent, with an effect on average fibre length. However, these are local symptoms of shortening to a certain size. The vast majority of the fibres remain intact, as in the virgin pulp. Therefore, it can be concluded that shortening of fibres can be tolerated to a certain extent if the overall change to the fibre mixture is small.

An image from the sample with an initial size of 1 × 1 mm shows many damaged fibres (Fig. 3c), i.e., disintegration into finer material. Therefore, excessive fragmentation of the fibrous material causes considerable damage to the fibrous fraction, and hence, deteriorates the potential of this fraction, which ultimately results in less desirable paper properties.

Based on the results obtained, the degree to which the shortened pieces of office waste paper can be converted into high-quality paper without any issues that reduce the value of the finished product can be predicted to a certain extent. From an ecological point of view, it is necessary to consider at the very least the issue of pulp freeness, and in turn, the energy needed for dewatering of the pulp. Excessive shortening of the paper samples worsens their dewatering capacity, as confirmed by the obtained results (Table 2). Therefore, the practice seeks to maintain a compromise and reduction of the refining range. Achieving efficiency in energy usage is considered the most cost-effective way to reduce CO_2_ emissions^65^. Therefore, by producing waste office paper without undue fragmentation in shredder (if data security issues need not be considered), the properties of recycled paper products can be improved and the energy consumption can be reduced concurrently. Thus, the recycling of shredded paper can be carried out in a more ecological, environmentally friendly, and economic manner.

## Conclusions

It was found in this study that the initial fibre length affects paper properties, the morphological characteristics of fibres, and the fine content of pulp, which in practice affects the cost of paper production. With other papermaking conditions held constant, paper samples produced from pulps with different initial fibre lengths showed various tensile properties. It is important to note that cutting paper into pieces with an area higher than 25 mm^2^ in the shredding process does not significantly influence the properties of pulp or the tensile properties of paper made from the pulp. Cutting samples into pieces smaller than 25 mm^2^, however, causes significant changes in the most important morphological parameters of the fibres and a sharp decrease in the dynamic and static strength properties of the paper with decreasing size of the shredded paper. Such a level of cutting is therefore unprofitable from a technological and an economic point of view because it required difficult pulp-dewatering processes and thus involves increased costs. The obtained results are therefore of great practical value because they demonstrate that the method of shredding paper in the shredder determines the processing potential of this pulp and the papermaking utility of the product. These conclusions can serve as a guide on how to responsibly shred paper, as well as to efficiently recycle fibres to manage biomass consumption.

## Data Availability

The datasets generated during and/or analysed during the current study are available from the corresponding author on reasonable request.
